# Effectiveness and safety of warm needle acupuncture on lumbar muscles strain: A protocol for systematic review and meta analysis

**DOI:** 10.1097/MD.0000000000024401

**Published:** 2021-03-05

**Authors:** Yashuang Huang, Cheng Cheng, Li Xie, Xinghui Zhu, Dongmei Chen, Cisong Cheng

**Affiliations:** School of Basic Medicine, Chengdu University of Traditional Chinese Medicine, China.

**Keywords:** acupuncture therapy, lumbar muscle strain, systematic review lumbar muscle strain, warm needle acupuncture

## Abstract

**Background:**

Lumbar muscle strain (LMS) is the most common orthopedic syndrome, with high incidence globally and lingering disease, which seriously affects patients’ work efficiency and quality of life. Warm needle acupuncture (WNA) is a treatment method combining acupuncture technology with warm and medicinal effect of moxibustion. It has outstanding curative effect and wide range of treatment, especially in the treatment of pain diseases. We aim to collect clinical evidence and demonstrate the efficacy and safety of WNA on LMS.

**Methods/design:**

We will search the following database sources for the randomized controlled trials: PubMed, Cochrane Library, Excerpta Medica Database (EMBASE), Web of Science, WHO International Clinical Trials Registry Platform (TCTRP), Chinese Biomedical Literature Database (CBM), Chinese National Knowledge Infrastructure Database (CNKI), Chinese Scientific Journals Database (VIP), and the Wanfang Database.

All randomized controlled trials of WNA for lumbar muscle strain (LMS) in the above database will be considered for inclusion, and high-quality articles will be screened for data extraction and analysis, to summarize the therapeutic effect of WNA on LMS patients.

**Result:**

This study will provide a rational synthesis of current evidences for warm needle acupuncture on lumbar muscle strain.

**Conclusion:**

The conclusion of this study will provide evidence to judge the effectiveness and safety of WNA on LMS.

**Trial registration:**

INPLASY2020120100 (DOI number: 10.37766/inplasy2020.12.0100).

## 1 Introduction

Lumbar muscle strain (LMS) is the most common orthopedic syndrome, with high incidence globally and lingering disease.^[1]^ With the change of modern lifestyle, long-term sitting or standing position, people lack of lumbar muscle exercise, the incidence of psoas muscle strain presents an increasing trend year by year.^[2]^ The onset of LMS is closely related to the special structure of the lumbar transverse process, especially the L3 transverse process, and the attached muscles. The main manifestation is recurrent soreness, swelling, or radiating pain in the waist on one or both sides, and even movement disorders. The pain can change with the degree of work or the weather, and it will be relieved after exercise.^[1]^ The recurrence rate of the disease is high, and the condition is lingering and difficult to heal. In severe cases, it can lead to loss, anxiety, depression, and other negative emotions, which seriously affect the work efficiency and quality of life of patients. Modern medical treatment of lumbar muscle strain usually adopts oral non-steroidal anti-inflammatory analgesics and surgery. The treatment costs are high and the long-term effect is not satisfactory. Therefore, acupuncture, massage, and other traditional Chinese medicine treatment methods have become the more commonly used treatment methods for the disease.

Warm needle acupuncture (WNA)^[3]^ is the most common and widely used therapy among traditional Chinese medicine (TCM) moxibustion. It is a kind of acupuncture technique combined with the warm and medicinal effect of moxibustion, and it has outstanding curative effect and a wide range of treatment. The combination of moxa and silver needle can increase its medicinal value. Under the action of acupuncture, the warm Qi of the drug can reach into the body, warm nourish the blood in the meridians and collaterals, and spread the cold clearing damp. WNA can promote local blood and lymph circulation, so that adhesion can be released, and then alleviate or eliminate edema. At the same time, warm acupuncture and moxibustion can also warm the meridians, smooth Qi and blood, nourish warm joints, bones and muscles, and reduce pain. It can also regulate the immune system to produce regulatory effects, so as to achieve the effect of harmonizing Yin and Yang, nourishing and dispelling evil.^[3]^ The indications of warm acupuncture and moxibustion are not limited to rheumatic diseases, mainly in a class of cold-related diseases, such as bone and joint disease,^[4,5]^ cold and painful skin, and abdominal distension,^[6]^ but expanded to the treatment of a variety of disease syndromes. It is widely used in low back pain,^[7]^ headache,^[8]^ dementia,^[9]^ stroke,^[10]^ gastritis,^[11]^ and other diseases. Taking the public concerns of opioid abuse and the favorable safety profile of acupuncture treatment into consideration, we would summarize clinical researches on WNA for lumbar muscle strain and findings of this review will be reliable within evidence of clinical studies. This review only focuses on the effects of warm acupuncture on insomnia rather than other effective treatments.

## 2 Methods

### 2.1 Study registration

We have been registered this review protocol on the International Platform of Registered Systematic Review and Meta-analysis Protocols (registration number, INPLASY2020120100). The protocol refers to the guidelines for the Preferred Reporting Items for Systematic Reviews and Meta-Analyses Protocols (PRISMA-P) which was stated on Feb 1st, 2019.

### 2.2 Inclusion criteria for study selection

#### 2.2.1 Types of studies

All available randomized controlled trials (RCTs) on WNA for lumbar muscle strain will be included. Others such as retrospective study, case report, review, and studies which uses inappropriate random sequence generation methods will be excluded.

#### 2.2.2 Types of participants

We will include studies on patients that have been diagnosed as LMS by clinicians based on Evidence-Based Clinical Guidelines for Multidisciplinary Spine Care: Diagnosis & Treatment of Low Back Pain,^[12]^ VA/DoD Clinical Practice Guideline: Diagnosis and Treatment of Low Back Pain and Criteria for Diagnosis,^[13]^ and Efficacy of TCM Diseases.^[14]^ There will be no restriction on age, gender, ethnicity, and profession.

The patient should meet the following diagnostic criteria:

1. Uninduced chronic weist and back pain is the main symptom, and the nature of weist and back pain is soreness.
2. There are fixed tender points near the muscle start and end points or neuromuscular junctions, and the pain will be reduced when the pain points are pressed again, which is different from deep bone disease.
3. Accompanied by symptoms of unilateral or bilateral muscle spasms.
4. Through film examination to exclude tumors, intervertebral disc herniation, recessive spina bifida, and third lumbar transverse process syndrome.

#### 2.2.3 Types of interventions

The purpose of the study is on clinical trials of WNA for lumbar muscle strain. Studies applied WNA in the experimental group will be included. WNA combined with other therapies will be excluded if the efficacy of WNA cannot be clarified in the combined therapy. The therapeutic intervention of controlled group can be other therapies such as conventional acupuncture, electro-acupuncture, auriculo-acupuncture, or pharmacological therapy.

#### 2.2.4 Types of outcome measures

##### 2.2.4.1 Primary outcome

The primary outcome is the variety of Oswestry Disability Index (ODI) and the visual analog scale (VAS). ODI^[15]^ is a common functional scale used in many studies to assess low back pain. The ODI questionnaire consists of 10 different questions. The questionnaire mainly focuses on pain (the degree of pain, the effect of pain on sleep), and individual functions (lifting objects, sitting, standing, walking), personal comprehensive ability (daily life, sexual life, social activities), each question has 6 options, the score is 0 to 5 points. The higher the score, the more severe the dysfunction. VAS^[16]^ is a commonly used pain scoring method in clinical practice. Before and after treatment, patients are asked to select the corresponding pain feeling on the scale of 0 to 10 (0 points means no pain, 10 points means severe pain) according to their own feelings. The higher the score, the more severe the dysfunction.

##### 2.2.4.2 Secondary outcomes

- Inflammatory factors: The change of inflammatory factors such as TNF-α, IL-6, CRP, etc. Studies^[2]^ have shown that LMS can cause the release of a large number of inflammatory factors. Inflammatory factors cause tissue edema to compress surrounding tissues, causing pain and tenderness. We observe the changes of inflammatory factors before and after treatment to judge the treatment effect.
- Activity of Daily Living Scales (ADL).
- JOA back pain evaluation questionnaire (JOABPEQ). JAOBPEQ is a new assessment standard established by the Japanese Orthopaedic Association to assess the quality of life of patients based on the principles of patient-based, multi-faceted, and scientific. It evaluates the patient's low back pain from 5 aspects: low back pain, waist function, walking ability, social life function, and mental health status, and is widely used in clinical practice.^[17]^
- Syndrome according to standards for assessing TCM.^[18]^
- Adverse events caused by WNA, such as dizziness, nausea, vomiting, weariness, etc.

### 2.3 Search methods for study identification

#### 2.3.1 Electronic searches

The following electronic databases will be searched from inception to NOV. 2020: 5 English databases (PubMed, Cochrane Library, Excerpta Medica Database [EMBASE], Web of Science, WHO International Clinical Trials Registry Platform [TCTRP]) and 4 Chinese databases (Chinese Biomedical Literature Database [CBM], Chinese National Knowledge Infrastructure Database [CNKI], Chinese Scientific Journals Database [VIP], and the Wanfang Database). To ensure literature saturation, we will scan the reference lists of included studies or relevant reviews identified through the search. We will also search the authors’ personal files to make sure that all relevant material has been captured. Various combinations of Medical Subject Headings and non-MeSH terms will be used, including lumbar muscle strain (LSM), WNA, and RTCs. The search terms with the equivalent English meaning will also be used in Chinese databases. The detailed search strategies in PubMed are provided in Table 1 and will be used similarly in other databases. Language will be restricted to Chinese and English.

**Table 1:** Search tactics for the PUBMED and web of science.

1. Search “acupuncture”[Mesh]OR “acupuncturetherapy”[Mesh]OR “bodyacupuncture” [Mesh]OR “manual acupuncture”
2. Search “moxibustion” [Mesh]OR “moxibustion therapy”[Mesh]
3. Search “lumbar muscle strain” [Mesh]OR “the strain of psoas muscle” [Mesh]OR “psoas muscle” [Mesh]OR “Strain of lumbar muscles” [Mesh]OR “lumbar muscle degeneration” [Mesh]OR “psoatic strain” [Mesh]OR “lumbar myofascitis”
4. Search (1 AND 2) AND 3
5. Search “warm needle acupuncture” [Mesh]OR “silver-needle warm acupuncture” [Mesh]OR “needle warming moxibustion” [Mesh]OR “moxibustion with warming needle” [Mesh]OR “needle warming moxibustion” [Mesh]OR “needle Warming Therapy” [Mesh]OR “warming acupuncture” [Mesh]OR “Wen Zhen”
6. Search 3 AND 5
7. Search 4 OR 6
8. Search “randomized controlled trial” [pt] OR “controlled clinic trial” [pt]OR “randomized” [tiab] OR “randomized allocation” [tiab] OR randomly [tiab] OR placebo [tiab] trial[tiab] OR groups [tiab]
9. Search 7 AND 8

#### 2.3.2 Searching other resources

Relevant systematic review or meta-analysis of RCTs will be electronically searched. Moreover, we will filter relevant medical journals and magazines to identify literature which is not included in the electronic databases.

#### 2.3.3 Search strategy

For example, the search strategy on PubMed are as follows: acupuncture (e.g., “acupuncture” or “acupuncture therapy” or “body acupuncture” or “manual acupuncture”); moxibustion (e.g., “moxibustion” or “moxibustion therapy”); warm needle acupuncture (e.g., “warm needle acupuncture” or “silver-needle warm acupuncture” or “needle warming moxibustion” or “moxibustion with warming needle” or “needle warming moxibustion” or “needle Warming Therapy” or “warming acupuncture” or “Wen Zhen”); lumbar muscle strain(e.g., “lumbar muscle strain” or “the strain of psoas muscle” or “psoas muscle” or “Strain of lumbar muscles” or “lumbar muscle degeneration” or “psoatic strain” or “lumbar myofascitis”). The complete PubMed and Web of science search strategy is summarized in Table 1.

If necessary, the strategy will be modified for use by other databases. Reference lists of related articles will also be checked for compliance.

### 2.4 Data collection and analysis

#### 2.4.1 Selection of studies

**Figure 1:**
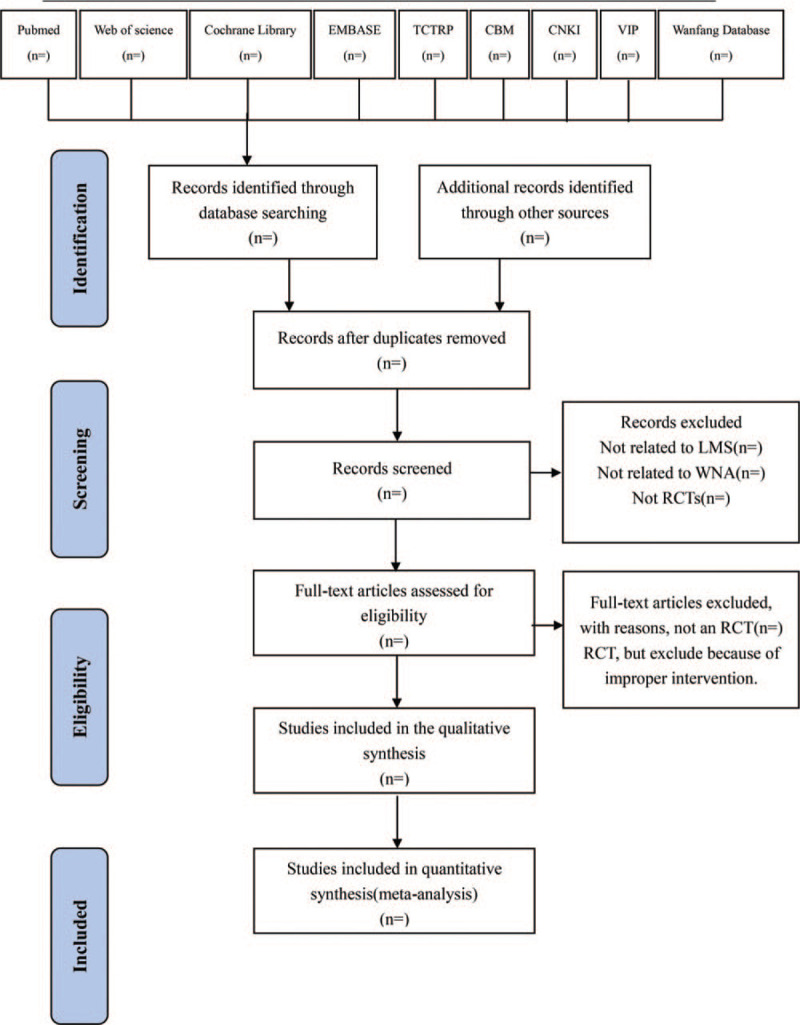
Flow diagram of study selection process. CBM = Chinese Biomedical Literature Database, CNKI = Chinese National Knowledge Infrastructure, EMBASE = Excerpta Medica Database, TCTRP = International Clinical Trials Registry Platform, VIP = Chinese Science and Technology Periodicals Database.

Two researchers will import the relevant studies obtained from the databases mentioned above into EndnoteX7, a literature management software. After removing duplicates, 2 researchers will independently evaluate the titles and abstracts of the searched studies and exclude the significantly unqualified literature. Later, the full text of the remaining studies will be read carefully and selected according to the inclusive criteria. Any different opinions generated between the 2 reviewers will be resolved through discussion. When consultation fails to reach an agreement, the third reviewer will step in and provide arbitration. The study selection procedure is shown in a flow chart according to PRISMA guidelines^[19]^ (Fig. 1).

#### 2.4.2 Data extraction and management

The data will be extracted by 2 independent researchers via a standardized data collection form, after reading the full texts of each included articles. The general information such as first author, country, time of publication, basic condition of the patient, design of study, sample size and number of dropouts, duration of follow-up, details of intervention, outcome measures, and adverse events associated with WNA will be extracted and recorded. We will contact the authors to request detailed information via e-mail or telephone, if the data are ambiguous or insufficient. Any divergence on data extraction will be discussed and judged by the 2 reviewers. The third reviewer will check the final results of the data extraction, provide arbitration for further disagreements.

#### 2.4.3 Assessment of risk of bias in included studies

Two authors independently assessed the methodological quality of each trial according to the standards advised by the Cochrane Handbook For Systematic Reviews of Interventions. Any disagreements were resolved by discussion and reached consensus through a third reviewer. We will assess the following domains: random sequence generation, allocation concealment, blinding to participants, personnel and outcome, incomplete outcome data, selective reporting, and other biases to evaluate the risk of bias of all included studies. Through our discussion, we will resolve any discrepancies in the assessment of risk of bias and consult an arbiter if it is necessary. Finally, we will divide the quality of the studies into 3 levels: “low risk of bias,” “high risk of bias,” and “unclear risk of bias.”

#### 2.4.4 Measures of treatment effect

Mean difference (MD) will be used to evaluate the extracted data for continuous variables, andrate ratio (RR) will be applied to analyze for dichotomous variables. The confidence intervals (CI) for both dichotomous and continuous variables will be set to 95%.

#### 2.4.5 Dealing with missing data

If we find insufficient or missing data during the statistical process, we will try to contact the corresponding author by telephone or e-mail. In addition, if the missing data really cannot be supplemented, we will analyze the potential impact of the missing data.

#### 2.4.6 Assessment of heterogeneity

Cochrane *Q* test is to be used to assess the existence of heterogeneity, and the extent of the heterogeneity will be quantified using the *I*^2^statistics. When the *I*^2^ value is <40% means might not be important; 30% < *I*^2^ < 60% means represent moderate heterogeneity; 50% < *I*^2^ < 90% means substantial heterogeneity; 75% < *I*^2^ < 100% means considerable heterogeneity. The random effects model will be selected and further subgroup analysis will be performed to investigate the possible causes of heterogeneity if the heterogeneity among trials is significant (*I*^2^ ≥ 50%). On the contrary, the fixed effect model will be chosen if an *I*^2^ values less than 50%.

#### 2.4.7 Assessment of reporting bias

In this analysis, once >10 trials are included, funnel plots could be used to test for reporting bias.

### 2.5 Statistical analysis

#### 2.5.1 Data synthesis

RevMan Software (V5.3, The Nordic Cochrane Centre, TheCochrane Collaboration, Copenhagen, Denmark) will conduct all data analysis. We will select a random effects model or fixed effects model to merge the primary and secondary outcome indicators in accordance with the results of heterogeneity test. If the heterogeneity is low (*I*^2^ < 50%), we will apply the fixed effects model for data synthesis, while the random effects model will be conducted of the significant heterogeneity (*I*^2^ ≥ 50%). It is considered that differences are statistically significant if the results of *Z* test show that *P* value is less than .05, and the 95% CI does not contain 0 (for continuous variables) or the 95% CI does not contain 1 (for dichotomous variables).

#### 2.5.2 Subgroup analysis

Once individual studies may consist of multiple treatment group, subgroup analysis will be performed to explain heterogeneity if possible. Factors such as following will be considered:

- Patients characteristics (age, sex, personal constitution, underlying diseases).
- Duration and frequency of therapy.

#### 2.5.3 Sensitivity analysis

A sensitivity analysis will be carry to identify the quality and robustness of the meta-analysis result once the outcome analyses involve a large degree of heterogeneity, according to sample size, methodological quality, and the effect of missing data.

#### 2.5.4 Assessment of reporting biases

When studying more than 10 trials, we will use a funnel chart to assess the existence of reporting bias. If the points of the funnel chart are scattered and asymmetric, we will consider that there is a reporting bias and the reliability is low. In the opposite case, it will be deemed that the reporting deviation does not exist and the results are reliable.

#### 2.5.5 Quality of evidence

This paper will use the evidence quality rating method to evaluate the results obtained from this analysis. We will assessed across the domains of risk of bias, consistency, directness, precision, and publication bias in accordance with the Recommendations Assessment, Development and Evaluation (GRADE)^[20]^ guidelines and rate it into 4 levels: high, moderate, low, or very low.

#### 2.5.6 Ethics and dissemination

There are no requirement of ethical approval and informed consent for all the data generated or analyzed during this study are publicly available.

## 3 Discussion

LMS is a common and frequently occurring disease in clinic. With the change of people's lifestyle, the incidence of LMS is increasing. Untreated treatment can lead to more serious problems such as disc herniation or prolapse, lumbar spinal stenosis, and acute lumbar sprain. Most commonly used western medicine is anti-inflammatory and analgesic, with poor efficacy and big side effects. Therefore, it is necessary to find a safe and effective treatment with few side effects and easy to accept.

WNA is an effective treatment method in traditional Chinese medicine. The combination of acupuncture and moxibustion has obvious immediate analgesic effect and lasting analgesic effect. WNA has good curative effect, simple operation, and easy acceptance. It can relieve pain, improve blood circulation, and stimulate the metabolism of local tissues. However, there is insufficient evidence on the effectiveness and safety of WNA, and its basic mechanism is still unknown. Therefore, it is necessary to conduct a systematic review and meta-analysis of the existing literature to evaluate the clinical efficacy and safety of WNA in LMS.

This will be the first systematic review and meta-analysis of WNA's treatment of LMS. First of all, the results of this review will provide objective statistical data for further WNA research. Secondly, the results will provide a reliable reference for clinicians and patients to use WNA to treat LMS. Third, the result may introduce alternative treatments for LMS to decision makers to reduce the burden on public health.

## Author contributions

**Conceptualization:** Yashuang Huang, Cheng Cheng.

**Data curation:** Yashuang Huang, Li Xie.

**Funding acquisition:** Cheng Cheng.

**Methodology:** Yashuang Huang, Cheng Cheng.

**Project administration:** Yashuang Huang, Xinghui Zhu, Dongmei Chen.

**Resources:** Li Xie, Dongmei Chen.

**Software:** Xinghui Zhu.

**Writing – original draft:** Yashuang Huang.

**Writing – review & editing:** Yashuang Huang, Cisong Cheng.
